# Overexpression of FcεRI on Bone Marrow Mast Cells, but Not MRGPRX2, in Clonal Mast Cell Disorders With Wasp Venom Anaphylaxis

**DOI:** 10.3389/fimmu.2022.835618

**Published:** 2022-02-25

**Authors:** Jessy Elst, Leander P. De Puysseleyr, Didier G. Ebo, Margaretha A. Faber, Athina L. Van Gasse, Marie-Line M. van der Poorten, Ine I. Decuyper, Chris H. Bridts, Christel Mertens, Michel Van Houdt, Margo M. Hagendorens, Luc S. De Clerck, Anke Verlinden, Katrien Vermeulen, Marie-Berthe Maes, Zwi N. Berneman, Peter Valent, Vito Sabato

**Affiliations:** ^1^ Department of Immunology, Allergology, Rheumatology and the Infla-Med Centre of Excellence, Faculty of Medicine and Health Sciences, University of Antwerp, Antwerp, Belgium; ^2^ Immunology, Allergology, Rheumatology, Antwerp University Hospital, Antwerp, Belgium; ^3^ Department of Immunology and Allergology, AZ Jan Palfijn Gent, Ghent, Belgium; ^4^ Department of Paediatrics and the Infla-Med Centre of Excellence, Faculty of Medicine and Health Sciences, University of Antwerp, Antwerp, Belgium; ^5^ Paediatrics, Antwerp University Hospital, Antwerp, Belgium; ^6^ Department of Haematology, Antwerp University Hospital, Antwerp, Belgium; ^7^ Department of Clinical Biology, Antwerp University Hospital, Antwerp, Belgium; ^8^ Division of Hematology and Hemostaseology, Department of Internal Medicine I, Medical University of Vienna, Vienna, Austria; ^9^ Ludwig Boltzmann Institute for Hematology and Oncology, Medical University of Vienna, Vienna, Austria

**Keywords:** human mast cell, mastocytosis, clonal mast cell disorder, MRGPRX2, FcεRI, flow cytometry, immunophenotype

## Abstract

**Background:**

Uncertainties remain about the molecular mechanisms governing clonal mast cell disorders (CMCD) and anaphylaxis.

**Objective:**

This study aims at comparing the burden, phenotype and behavior of mast cells (MCs) and basophils in patients with CMCD with wasp venom anaphylaxis (CMCD/WVA^+^), CMCD patients without anaphylaxis (CMCD/ANA^-^), patients with an elevated baseline serum tryptase (EBST), patients with wasp venom anaphylaxis without CMCD (WVA^+^) and patients with a non-mast cell haematological pathology (NMHP).

**Methods:**

This study included 20 patients with CMCD/WVA^+^, 24 with CMCD/ANA^-^, 19 with WVA^+^, 6 with EBST and 5 with NMHP. We immunophenotyped MCs and basophils and compared baseline serum tryptase (bST) and both total and venom specific IgE in the different groups. For basophil studies, 13 healthy controls were also included.

**Results:**

Higher levels of bST were found in CMCD patients with wasp venom anaphylaxis, CMCD patients without anaphylaxis and EBST patients. Total IgE levels were highest in patients with wasp venom anaphylaxis with and without CMCD. Bone marrow MCs of patients with CMCD showed lower CD117 expression and higher expression of CD45, CD203c, CD63, CD300a and FcεRI. Within the CMCD population, patients with wasp venom anaphylaxis showed a higher expression of FcεRI as compared to patients without anaphylaxis. Expression of MRGPRX2 on MCs did not differ between the study populations. Basophils are phenotypically and functionally comparable between the different patient populations.

**Conclusion:**

Patients with CMCD show an elevated burden of aberrant activated MCs with a significant overexpression of FcεRI in patients with a wasp venom anaphylaxis.

## Introduction

Clonal mast cell disorders (CMCD) originate from morphological and immunophenotypical aberrant mast cells (MCs) that proliferate abnormally and accumulate in different organs (1). In most cases, this abnormal proliferation is driven by a somatic gain-of-function mutations of the KIT receptor, also known as CD117. In the majority of cases this is the KIT D816V mutation (2, 3).

Anaphylaxis is one of the cardinal features of non-advanced CMCD and occurs approximately in up to 50% of the patients (4, 5). Despite significant progress in our understandings of the molecular mechanisms and pathophysiology behind anaphylaxis, predicting anaphylactic risk in CMCD remains difficult. The prevalence of anaphylaxis in patients with CMCD is estimated to be a 100 to 1,000 times higher than in patients without a CMCD (4, 6). However, some patients with a CMCD present with one or multiple episodes of anaphylaxis while others can pass a lifetime without an anaphylactic event. Likely, the reasons are multifactorial and relate to, among others, the MC burden that is best reflected by baseline serum tryptase (bST). Patients with CMCD have a higher MC burden and a bST of >20 ng/mL is a minor criterion for the diagnosis (7). Although increased levels of bST have been associated with the risk of severe anaphylaxis (8), this association is not absolute. A study by van Anrooij et al., showed that the prevalence of Hymenoptera venom anaphylaxis in patients with systemic mastocytosis (SM) increased up until the 6^th^ decile of bST (28 ng/mL) but decreased with higher levels of bST (9).

However, the MC burden alone cannot entirely explain the increased risk of anaphylaxis in CMCD. Other contributing factors are the perivascular localization of MCs and the intrinsic (partially elusive) MC characteristics (10). A recently described, IgE-independent mechanism, is the activation of MCs through the Mas-related G protein-coupled receptor-X2 (MRGPRX2). This receptor can directly be engaged by various agonists like quinolones, opiates, substance P, neuromuscular blocking agents and mastoparan, a vespid venom component (11–13). Some have hypothesized that this receptor might attribute to wasp venom anaphylaxis in patients with mastocytosis (13). Furthermore, basophils can play a non-redundant role in anaphylaxis, especially wasp venom anaphylaxis (14) and it is under debate whether mature basophils express the typical MC receptor CD117 (15–19).

The aim of this study is to compare the burden, phenotype and functional behavior of MCs and basophils in patients with a CMCD who experienced wasp venom anaphylaxis, patients with a CMCD but without anaphylaxis, patients an elevated baseline serum tryptase (bST >11.4 ng/mL) without evidence of a CMCD (EBST), patients with wasp venom anaphylaxis but without a CMCD and patients with a non-mast cell related haematological pathology (NMHP).

## Methods

### Study Population

**Figure 1:**
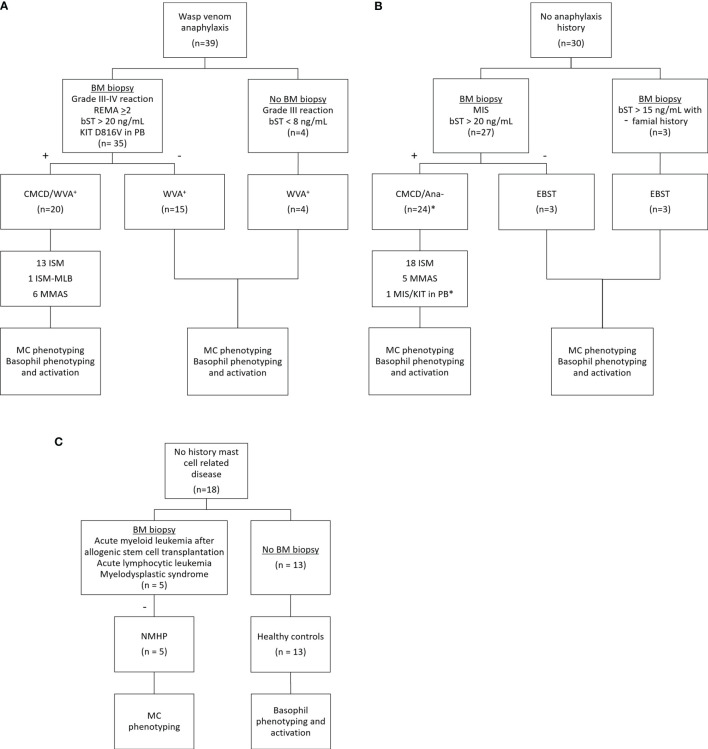
Inclusion of study patients. **(A)** Patients with wasp venom anaphylaxis received a bone marrow biopsy if they had clinical and laboratory findings suggestive for CMCD (n=35). The four other patients with wasp venom anaphylaxis that did not receive a bone marrow biopsy had no clinical and/or laboratory finding suggestive for CMCD. **(B)** Patients without anaphylaxis received a bone marrow biopsy based on their clinical and/or laboratory finding suggestive for CMCD (n=27). Three patients with a slightly elevated baseline tryptase and with a familial history received also a bone marrow biopsy. One patient, indicated with the asterisk (*) refused a bone marrow biopsy but is included in the CMCD/ANA- group based on MIS and a positive c-kit D816V mutation in peripheral blood. **(C)** Patients with no history of a mast cell related disease received a bone marrow based on other clinical implications. CMCD/WVA+ = Patients with a clonal mast cell disorder and wasp venom anaphylaxis. WVA+ = Patients with wasp venom anaphylaxis and without clonal mast cell disorder. CMCD/ANA- = Patients with a clonal mast cell disorder without anaphylaxis. EBST = patients with an elevated baseline serum tryptase without anaphylaxis and a clonal mast cell disorder. NMHP = Patients with a non-mast cell related haematological pathology. ISM, indolent systemic mastocytosis; ISM-MBL, indolent systemic mastocytosis with an associated monoclonal B-cell lymphocytosis; MMAS, monoclonal mast cell activation syndrome; MIS, mastocytosis in the skin; CMCD, clonal mast cell disorder; BM, bone marrow; PB, peripheral blood; bST, baseline serum tryptase; +, positive bone marrow biopsy investigations; -, negative bone marrow biopsy investigations.

**Figure 1** shows the patient inclusion. This study included 39 patients with a history of a grade III-IV according to Muller (20) anaphylaxis due to wasp venom. The diagnosis of wasp venom anaphylaxis was confirmed by positive skin testing and specific IgE (sIgE) to wasp venom, according to the EAACI guidelines (21). Based on clinical parameters [severe hypotension without mucocutaneous symptoms and/or mastocytosis in the skin (MIS) (22)] and/or laboratory findings (bST > 20 ng/mL and/or KIT D816V mutation in peripheral blood), 35 patients underwent a bone marrow (BM) biopsy. For the remaining 4 patients, the clinical and laboratory features were not suggestive for a CMCD (Grade III anaphylaxis with a bST below 8 µg/mL) and were therefore not subjected to a BM biopsy (5). After diagnostic work-up, twenty patients were included in the CMCD group and 19 in the wasp venom anaphylaxis group without a CMCD, the latter including the 4 patients without a bone marrow biopsy. Diagnosis of a CMCD was established according to the WHO criteria (7, 23). Of the 20 patients with a CMCD, 13 were diagnosed with an indolent systemic mastocytosis (ISM), 1 patient had a ISM with an associated haematological neoplasm (ISM-Monoclonal B-cell lymphocytosis) and 6 patients had monoclonal mast cell activation syndrome (MMAS) (**Table 1**). The remaining 15 patients had no signs of clonality or aberrancy in BM histology and aspirate and were therefore included in the wasp venom anaphylaxis group without a CMCD.

**Table 1:** Demographical data and laboratory values.

	*CMCD/WVA^+^*	*CMCD/ANA^-^*	*WVA^+^*	*EBST*	*HC*	*NMHP*
**Number**	20	24	19	6	13	5
**SM subtypes**	13 ISM	18 ISM				
	1 ISM-MBL	6 MMAS				
	6 MMAS					
**Sex (M/F)**	10/10	9/15	13/6	3/3	6/7	3/2
**Median age (range)**	61	43	60	59	29	63
	(35-76)	(19-75)	(37-76)	(42-72)	(21-55)	(25-68)

CMCD/WVA+ = Patients with a clonal mast cell disorder and wasp venom anaphylaxis. CMCD/ANA- = Patients with a clonal mast cell disorder without anaphylaxis. WVA+ = Patients with wasp venom anaphylaxis and without clonal mast cell disorder. EBST = patients with an elevated baseline serum tryptase without anaphylaxis. HC, Healthy controls; NMHP = patients with a non-mast cell related haematological pathology ISM, indolent systemic mastocytosis; ISM-MBL, indolent systemic mastocytosis with an associated monoclonal B-cell lymphocytosis; MMAS, monoclonal mast cell activation syndrome.

In addition, 27 patients without anaphylaxis but with other clinical (MIS) and/or laboratory (baseline tryptase > 20 ng/mL) suggestive presentation for a CMCD were included. These patients, underwent BM biopsy to evaluate the presence of an underlying CMCD. After diagnostic work-up, 24 patients were included in the group of CMCD patients without anaphylaxis. Of the 24 patients with a CMCD patients without anaphylaxis, 18 were diagnosed as ISM and 5 had MMAS. One patient presented with MIS and had a positive KIT DV816V mutation in PB but refused the BM biopsy. This patient was included in the CMCD patients without anaphylaxis group because the adult onset of MIS and the presence of KIT D816V mutation in peripheral blood (PB) (24). Three of the 27 patients had no signs of a CMCD after full diagnostic work-up but had only an elevated bST. Additionally, three other patients received a bone marrow biopsy based on a slightly elevated baseline tryptase (>15 ng/mL) with a familial history of elevated tryptase. All had negative bone marrow investigation for CMCD. The 6 patients with negative bone marrow investigations also had first degree family members with an elevated tryptase which is suggestive of hereditary alfa-tryptasemia. However, we did not have access to the assay to determine TPSAB1 copy numbers. Therefore, we labelled this group as elevated baseline serum tryptase (EBST) (**Table 1**).

Finally, five patients with a non-mast cell related haematological pathology (NMHP) who underwent BM biopsy for diagnosis or follow-up of treatment were included as control group for the MC experiments. These 5 patients suffered from acute myeloid leukemia treated with allogenic stem cell transplantation (3), acute lymphocytic leukemia (1) and myelodysplastic syndrome (1). For the basophil experiments, 13 healthy controls (HC) were included who had no history of allergies and were not on antihistamines, anti-leukotrienes, omalizumab, steroids or other immunomodulating medication. Approval for this study was obtained from the local ethical committee (B300201836890). Patients and HC signed an informed consent. Further demographic details of the study population can be found in **Table 1**.

### Baseline Serum Tryptase Allergy Work-Up and KIT D816V Mutation

Basal serum tryptase and total IgE (tIgE) were quantified by the FEIA ImmunoCAP (Phadia Thermo Fisher Scientific, Uppsala, Sweden). In addition, sIgE to wasp (i3), recombinant (r) Ves v 1 and rVes v 5 were determined in patients with WVA. Ratios for sIgE-to-tIgE were calculated.

KIT D816V mutation in PB and BM was determined, as described earlier (24), using digital droplet PCR (Bio-Rad, Hercules, USA). Quantification of the KIT D816V allele burden in the BM cells was available in 14 patients with a CMCD with wasp venom anaphylaxis and 13 patients with a CMCD patients without anaphylaxis with a sensitivity of at least 0.01% positive alleles.

### Sampling

Phenotyping studies of bone marrow MCs were performed from 2-6 mL of EDTA BM collected from patients. Bone marrow aspiration was performed in posterior iliac spina, using an 11- to 8-G biopsy needle. Besides, 10 mL of EDTA and heparinized whole blood were collected respectively for immunophenotyping and activation studies of basophils. Bone marrow and blood samples were analyzed within 4 hours after collection.

### Phenotyping of Mast Cells and Basophils; Functional Studies of Basophils

#### Antibodies for Staining

Anti-human CD45-PerCP (clone H130), anti-human MRGPRX2-PE (clone K125H4), anti-human CD63-AF488 (clone H5C6) were purchased from Biolegend (San Diego, CA, USA). Anti-human CD117-APC (clone 104D2), anti-human CD63-PE (clone H5C6) from BD Bioscience (Erembodegem, Belgium). Anti-human CD203c-PeCy7 (clone NP4D6) and anti-human basophil granules (clone 2D7) were purchased from eBioscience (San Diego, CA, USA). Anti-human CD300a-PE (clone E59.126) and anti-human CD25-FITC (clone B1.49.9) from Beckman Coulter (Brea, CA, USA). Anti-human IgE (clone GE-1, Sigma Aldrich, St-Louis, MO, USA) was labelled with Alexa Fluor 405 (Molecular Probes, Invitrogen, Paisley, UK). Anti-human FcεRI-PE (clone AER-37) and anti-human FcεRI (clone, AER-375(CRA1) labelled with Alexa Fluor 700 (Phadia Thermo Fisher Scientific).

#### Reagents for Basophil Activation Experiments

Anti-IgE (0.5mg/mL, clone G7-18, BD Bioscience), f-Met-Leu-Phe (fMLP, 5mg/mL, Sigma-Aldrich), stem cell factor (SCF, Miltenyi Biotec, Bergisch Gladbach, Germany) were used.

#### Immunophenotyping of Resting MC

The BM samples were incubated with lysis buffer (for 10 min-1 h) (Biolegend) at room temperature. Afterwards, cells were washed twice, first with lysis buffer and secondly with PBS-BSA 0.5% (Sigma-Aldrich) and stained with anti-human CD45-PerCP, anti-human CD117-APC, anti-human CD203c-PECy7, anti-human FcεRI-PE, anti-human MRGPRX2-PE, anti-human CD300a-PE, anti-human CD63-PE and anti-human CD25-FITC. Cells were stained for 15 min at 4°C in the dark and washed with PBS-BSA 0.5% and suspended in PBS supplemented with 0.1% paraformaldehyde (Biolegend).

#### Immunophenotyping of Resting and Activated Basophils

Phenotyping of resting basophils in EDTA blood was based on staining with anti-human IgE-AF405, anti-human CD63-AF488, anti-human CD203c-PECy7, anti-human CD300a-PE, anti-basophil granules and anti-human FcεRI-AF700. For activation experiments, heparinized whole blood basophils were incubated (37°C, 20 min) with 200 µL anti-IgE or fMLP at the optimal concentrations of respectively 5 µg/mL, 0.5 µg/mL. We also investigated whether basophils expressed SCF receptor (CD117) and if engagement with its ligand SCF would alter the activation. Therefore, we phenotyped resting basophils with anti-human CD117-APC and performed functional experiments with SCF at concentrations of 50, 100 or 200 ng/mL. Of note, the basophilic lineage was confirmed by positivity to the monoclonal antibody that binds to a basophil specific protein localized in the secretory granules (clone 2D7) (**Figure 7B**) (25). Besides, co-stimulation with SCF for both activators (fMLP and anti-IgE) were performed with the earlier mentioned end concentrations. For the functional experiments, cells were stained with anti-human IgE-AF405, anti-human CD63-FITC, anti-human CD203c-PECy7 and anti-human CD117-APC. Cells were stained for 20 min at 4°C in the dark, lysed and fixated with Lyse/Fix (BD bioscience) and washed and suspended in PBS supplemented with 0.1% sodium azide (PBS-NaN_3_).

#### Flow Cytometric Analysis

Flow cytometric analysis was performed on a calibrated FACSCanto II flow cytometer (BD Immunocytometry Systems, San Jose, CA) equipped with three lasers (405 nm, 488 nm and 633 nm). Correct compensation settings were performed using BD CompBeads (BD Biosciences). Flow cytometric data were analyzed using Kaluza Analysis 2.1 software (Beckman Coulter) or FCS-express 6 flow research edition (DeNovo software, Glendale, CA, USA). **Supplementary Figure 1**, shows the gating strategy of basophils and bone marrow mast cells. Fluorescence minus one (FMO) samples was used to set a marker between positive and negative cells according to the 99^th^ percentile. As shown in **Supplementary Figure 2**, the markers were set individually. Basophils were selected as low side scatter and IgE^+^/CD203c^+^; MCs were selected as CD45^+^/CD117^+^/CD203c^+^ cells. Density measurements were performed using standardized fluorospheres (SPHERO Ultra Rainbow Calibration particles, Spherotech, Lake Forest, IL, USA) as described by the manufacturer. Results were expressed as the Molecules of Equivalent Soluble Fluorochrome (MESF). At least 1,000 basophils were counted. We counted a minimum of 25 MCs in BM samples where at least 3.10^6^ CD45^+^ cells were collected. The percentage of MCs was calculated by dividing the number of MCs through the total number of CD45^+^ cells. The basophil count was calculated using AccuCheck counting beads (Thermofisher Scientific) according to the manufacturer’s instructions.

### Statistical Analysis

All results were expressed as median (range). A Kruskal-Wallis test was performed and where appropriate followed by non-parametric Mann-Whitney U tests. *P*-values ≤ 0.05 were considered significant. Statistical significance in the functional experiments was calculated between the different groups and for the different stimulation conditions within the groups (anti-IgE, fMLP, SCF, anti-IgE+SCF, fMLP+SCF) by a Friedman test. Correlation between different parameters was calculated using linear regression. Results were analysed using PRISM 8 (GraphPad Software, San Diego, CA, USA) and JMP (SAS software, Cary, NC, USA).

## Results

### Baseline Serum Tryptase Is Increased in CMCD Patients and the KIT D816V Allele Burden Is Comparable Between CMCD Patients With and Without Anaphylaxis

**Figure 2:**
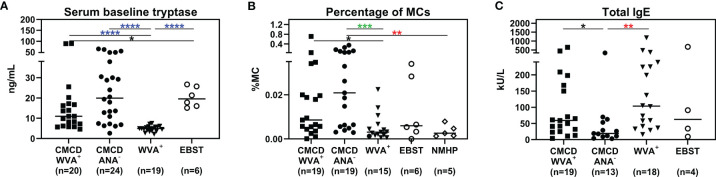
Serum baseline tryptase **(A)**, percentage of mast cells in the bone marrow **(B)** and total IgE **(C)**. **(A)** Median bST of patients with wasp venom anaphylaxis (WVA^+^) is significantly lower than in patients with a CMCD and wasp venom anaphylaxis (CMCD/WVA^+^), patients with a CMCD but without anaphylaxis (CMCD/ANA^-^) and a patients with elevated baseline serum tryptase without anaphylaxis (EBST). **(B)** The percentages of MCs were significantly lower in patients with wasp venom anaphylaxis (WVA^+^) compared to patients with a CMCD with wasp venom anaphylaxis (CMCD/WVA^+^) and a CMCD patients without anaphylaxis (CMCD/ANA^-^). **(C)** Total IgE was significantly lower in patients with a CMCD without anaphylaxis (CMCD/ANA^-^) as compared to patients with a CMCD with wasp venom anaphylaxis (CMCD/WVA^+^) and patients with wasp venom anaphylaxis (WVA^+^). NMHP = Patients with a non-mast cell related haematological pathology. Connecting lines represent significant differences *p < 0.05 (black), **p < 0.01 (red), ***p < 0.001 (green), ****p < 0.0001 (blue). Non-significant differences are not indicated.

**Figure 3:**
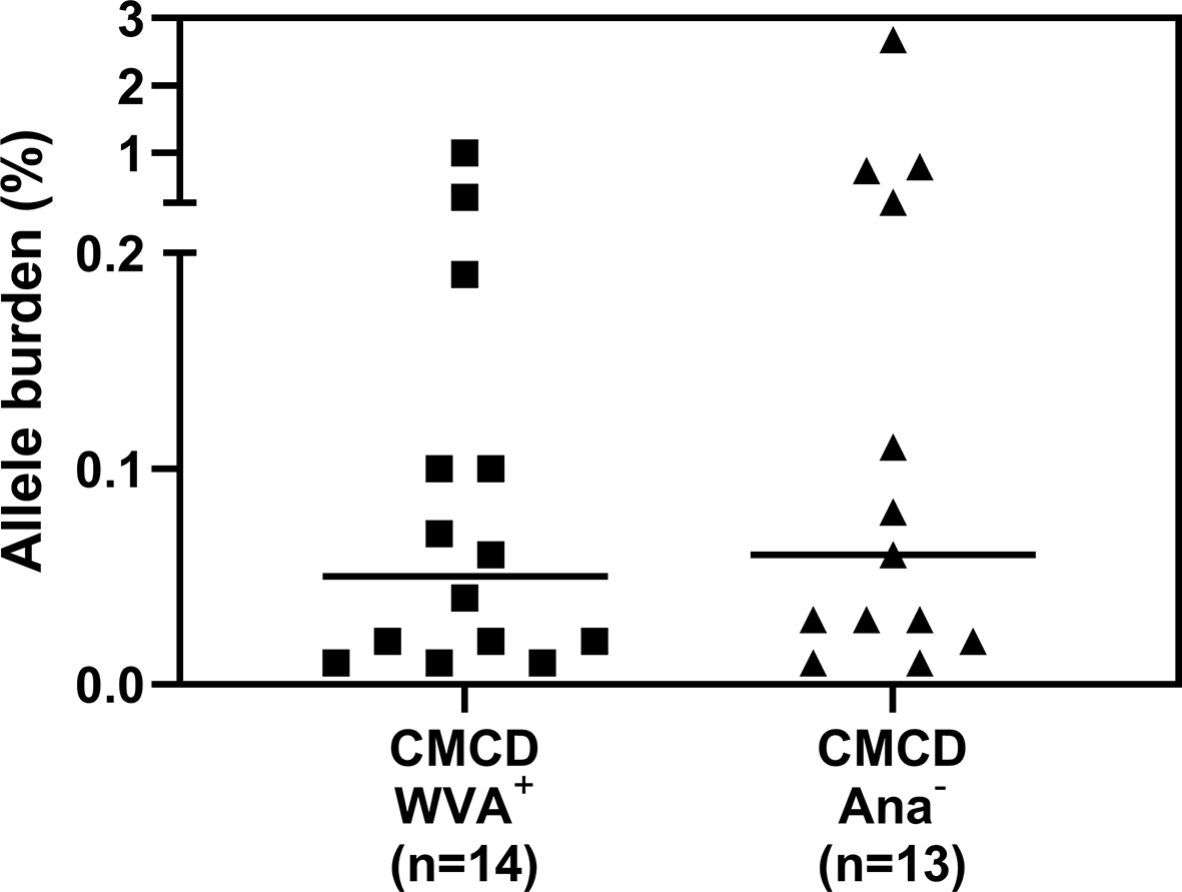
Allele burden of KIT D816V mutation in CMCD patients with and without anaphylaxis. There is no significant difference in allele burden between CMCD patients with wasp venom anaphylaxis (CMCD/WVA^+^) and CMCD patients who had no history of anaphylaxis (CMCD/ANA^-^).

Median bST of patients with wasp venom anaphylaxis was 4.9 (2.2-7.5) ng/mL and was significantly lower than in patients with a CMCD and wasp venom anaphylaxis (11 ng/mL, 4.6-89.1 ng/mL), patients with a CMCD but without anaphylaxis 19.6 (2.6-64.8) ng/mL and a EBST 19.55 (15.1-26.7) ng/mL (**Figure 2A**). As shown in **Figure 2B**, percentages of MCs were significantly lower in patients with wasp venom anaphylaxis compared to patients with a CMCD with wasp venom anaphylaxis and a CMCD patients without anaphylaxis. A KIT D816V mutation was present in 16 of the 20 patients with a CMCD with wasp venom anaphylaxis (80%) with an allele burden of 0.05% (0.01-1%). In these subpopulation, a KIT D816V mutation was present in all the 13 patients with ISM (100%) and in the patient with ISM with an associated haematological neoplasm (100%), of the patients with MMAS 2 of the 6 patients presented the mutation (33%). Regarding to the patients with CMCD without anaphylaxis, a KIT D816V mutation was present in 20 of the 24 patients with a CMCD without anaphylaxis (83%) with an allele burden of 0.06% (0.01-2.68%). A KIT D816V mutation was present in 17 of the 19 patients with an ISM (89%) and in the 3 of the 5 patient with MMAS (60%). As shown in **Figure 3**, the allele burden was similar in both patient groups (CMCD with wasp venom anaphylaxis and CMCD patients without anaphylaxis). As shown in **Figure 2C** and **Table 1**, tIgE was significantly lower in patients with a CMCD without anaphylaxis as compared to patients with a CMCD with wasp venom anaphylaxis and patients with wasp venom anaphylaxis. In the patients with wasp venom anaphylaxis (with and without a CMCD), there was no significant difference between sIgE and the sIgE-to-tIgE ratios (**Supplementary Figure 3**).

### MCs From CMCD Patients With Anaphylaxis Show Higher Expression of FcεRI, but Not MRGPRX2

**Figure 4:**
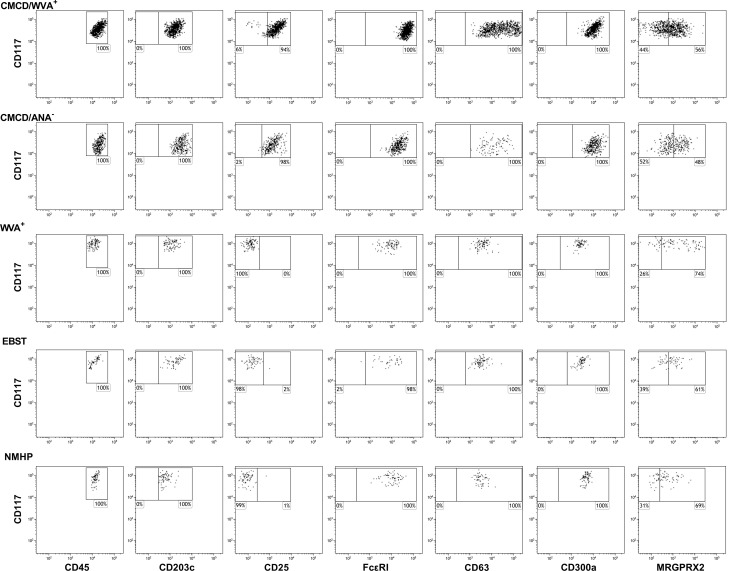
Representative flow cytometric plots of the staining of membrane markers on bone marrow mast cells. Mast cells were defined as CD45^+^CD117^+^CD203c^+^. All the gated MCs were positive for FcϵRI, CD300a and CD63. MRGPRX2 expression is only partially expressed on the MCs. CMCD/WVA^+^ = Patients with a clonal mast cell disorder and wasp venom anaphylaxis. CMCD/ANA^-^ = Patients with a clonal mast cell disorder without anaphylaxis. WVA^+^ = Patients with wasp venom anaphylaxis and without clonal mast cell disorder. EBST = patients with an elevated baseline serum tryptase without anaphylaxis and NMHP = Patients with a non-mast cell related haematological pathology.

**Figure 5:**
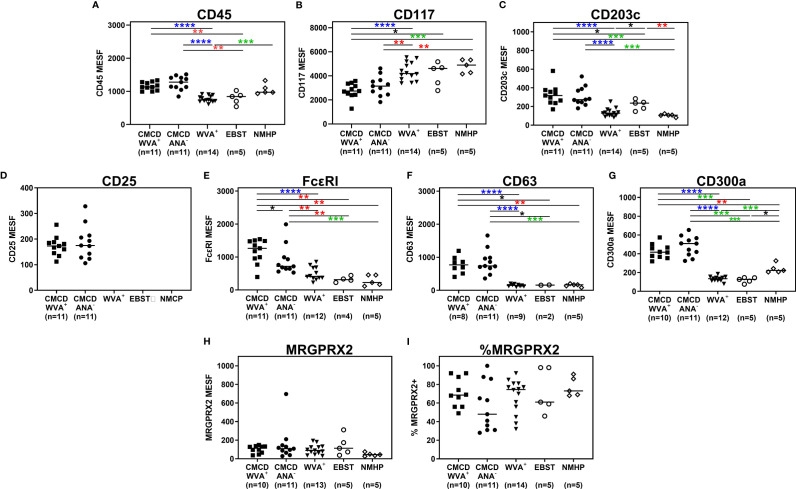
Expression of immunophenotypic markers on bone marrow mast cells. **(A)** CD45 was significantly overexpressed on MCs from CMCD/WVA^+^ and CMCD/ANA^-^ patients as compared to WVA^+^, EBST and NMHP patients. **(B)** MCs of CMCD/WVA^+^ and CMCD/ANA^-^ patients had a significantly lower expression of CD117 on the cell surface compared to WVA, EBST and NMHP patients. **(C-G)** CD203c, CD63, CD300a and FcϵRI were significantly overexpressed on MCs from CMCD/WVA^+^ and CMCD/ANA^-^ patients as compared to WVA^+^, EBST and NMHP patients. FcεRI was significantly higher expressed on MCs CMCD/WVA^+^ patients compared to CMCD/ANA^-^ patients. **(H, I)** There is no significant difference in expression of MRGPRX2, neither in percentage **(H)** nor in density **(I)**, between the various patient groups. **(A-H)**, the density is expressed as MESF/cell CMCD/WVA^+^ = Patients with a clonal mast cell disorder and wasp venom anaphylaxis. CMCD/ANA^-^ = Patients with a clonal mast cell disorder without anaphylaxis. WVA^+^ = Patients with wasp venom anaphylaxis and without clonal mast cell disorder. EBST = patients with an elevated baseline serum tryptase without anaphylaxis and NMHP = Patients with a non-mast cell related haematological pathology. Connecting lines represent significant differences *p < 0.05 (black), **p < 0.01 (red), ***p < 0.001 (green), ****p < 0.0001 (blue). Non-significant differences are not indicated.

**Figure 4** show representative plots of the phenotypical analyses of membrane markers on bone marrow MCs. All the gated MCs were positive for FcεRI, CD300a and CD63. **Figure 5** shows the individual data of the expression of the different surface markers. MCs of patients with a CMCD with wasp venom anaphylaxis and a CMCD patients without anaphylaxis had a significantly lower expression of CD117 on the cell surface compared to patients with wasp venom anaphylaxis, EBST and NMHP patients. CD45, CD203c, CD63, CD300a and FcεRI were significantly overexpressed on MCs from patients with a CMCD with wasp venom anaphylaxis and CMCD without anaphylaxis as compared to patients with wasp venom anaphylaxis, EBST and NMHP patients. FcεRI was significantly higher expressed on MCs of patients with a CMCD and a wasp venom anaphylaxis compared to patients with a CMCD without anaphylaxis. Within the subpopulations, there was no correlation between the density of the FcεRI and the titer of tIgE, v-sIgE, sIgE-to-tIgE ratio and bST (data not shown). With respect to the expression of MRGPRX2, no significant difference was seen, neither in percentage nor in density, between the various patient groups (**Figures 5H, I**). In all patients with a CMCD, MCs displayed CD25 but no difference was seen between patients with or without WVA. Of note, in our population there were no phenotypical differences between CMCD patients diagnosed with ISM or with MMAS (data not shown).

**Figure 6:**
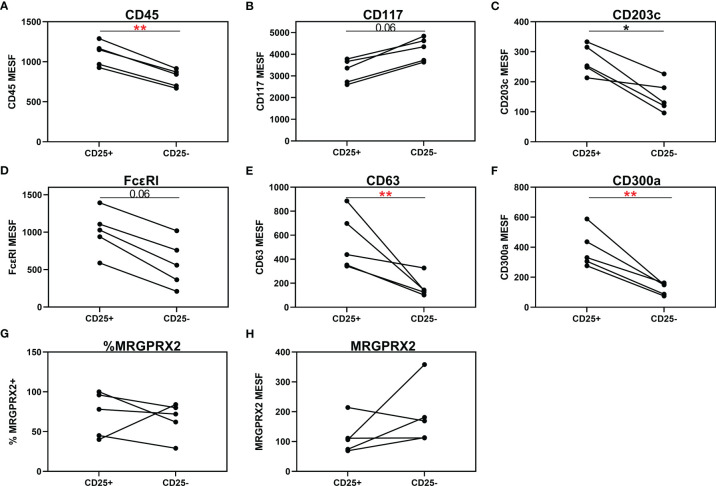
Individual comparison of aberrant (CD25^+^) and non-aberrant (CD25^-^) MC population in patients with both subpopulations. **(A)** CD45 is significantly overexpressed on the aberrant MCs. **(B)** Aberrant MCs had a lower expression of CD117. **(C–F)** CD203c, FcεRI, CD63 and CD300a were significantly overexpressed on the aberrant MCs. **(G, H)** There is no difference in expression of MRGPRX2, neither in percentage **(G)** nor in density **(H)**, between aberrant and non-aberrant MCs. **(A–F, H)**, the density is expressed as MESF/cell. Connecting lines represent significant differences *p < 0.05 (black), **p < 0.01 (red). Non-significant differences are not indicated.

Three of the 20 patients with a CMCD with wasp venom anaphylaxis and 2/24 patients with a CMCD patients without anaphylaxis, displayed two subpopulations, an aberrant (CD25 positive) and a non-aberrant (CD25 negative) MC population. As shown in **Figure 6**, intra-individual comparison showed that the aberrant MCs had a lower expression of CD117. In contrast, CD45, CD203c, FcεRI, CD63 and CD300a were significantly overexpressed on the aberrant MCs. Regarding the expression of MRGPRX2, no difference was seen, neither in percentage nor in density, between aberrant and non-aberrant MCs.

### Basophil Phenotype and Functionality Is Comparable in All Patients’ Populations

**Figure 7:**
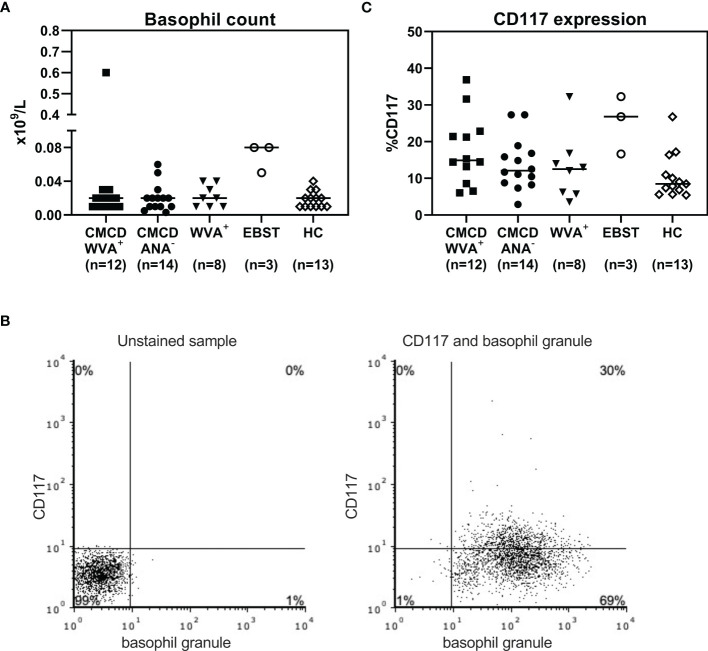
Basophil count **(A)** and expression of CD117 on the surface of basophils **(C)**. **(A)** All study populations had comparable numbers of circulating basophils. **(B)** l A representative plot of CD117 staining in basophils including staining with basophil specific granule (clone 2D7). **(C)** There are no significant differences in CD117 expression on the surface basophils between the different groups. CMCD/WVA^+^ = Patients with a clonal mast cell disorder and wasp venom anaphylaxis. CMCD/ANA^-^= Patients with a clonal mast cell disorder without anaphylaxis. WVA^+^ = Patients with wasp venom anaphylaxis and without clonal mast cell disorder. EBST = patients with an elevated baseline serum tryptase without anaphylaxis. HC = Healthy controls.

**Figure 8:**
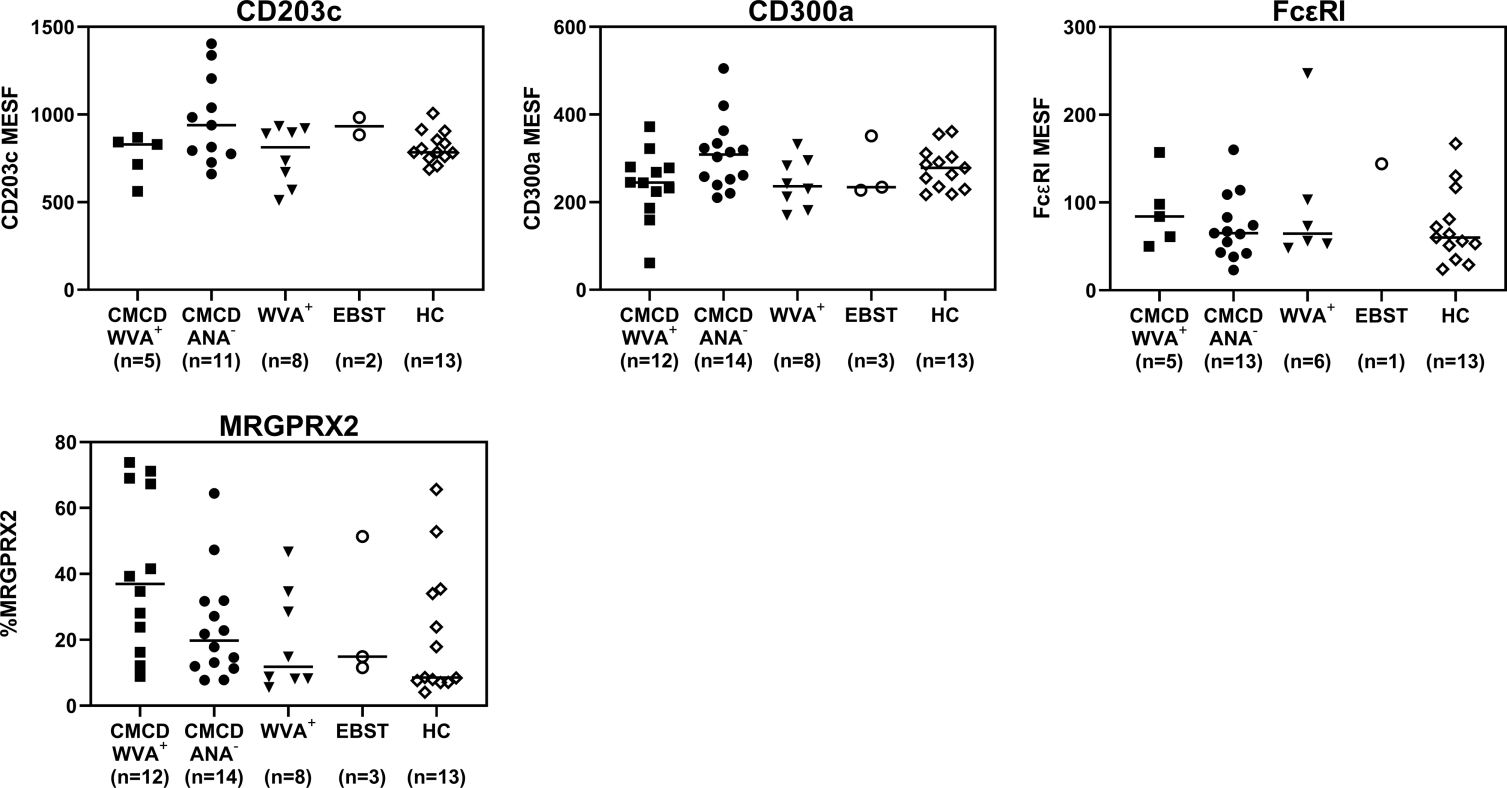
Expression of CD203c, CD300a, FcϵRI and MRGPRX2 on the surface of peripheral blood basophils. There are no significant differences in expression of CD203c, CD300a, FceRI and MRGPRX2 on the surface of basophils. CMCD/WVA+ = Patients with a clonal mast cell disorder and wasp venom anaphylaxis. CMCD/ANA-= Patients with a clonal mast cell disorder without anaphylaxis. WVA+ = Patients with wasp venom anaphylaxis and without clonal mast cell disorder. EBST = patients with an elevated baseline serum tryptase without anaphylaxis. HC = Healthy controls.

As shown in **Figure 7A**, all study populations had comparable numbers of circulating basophils. **Figure 7B** shows a representative plot of CD117 staining in basophils. Of note, the basophilic lineage was confirmed by positivity the monoclonal antibody that binds to a basophil specific protein localized in the secretory granules (clone 2D7) (25). As shown **Figure 7C**, between 3-37% of basophils expressed CD117 on their cell surface, however no difference was found between the different groups. Incubation of the basophils with SCF induced a decrease of surface CD117, irrespective of the included patient population (data not shown). In addition, no significant differences were found in density of CD203c, CD300a or FcεRI on the cell membrane of basophils between the different groups (**Figure 8**).

Functionally, as shown in **Supplementary Figure 4**, no difference was found between the different study population (upregulation of activation markers, CD63 and CD203c). Furthermore, coincubations with various concentrations of SCF did not alter responsiveness of the cells neither to anti-IgE, nor to fMLP.

## Discussion

Despite significant progress in our understanding of the molecular mechanism behind anaphylaxis, it remains difficult to predict the risk for anaphylaxis in patients with or without a CMCD. Probably, this risk relates to the MC burden, intrinsic characteristics of MCs and basophils and the perivascular localization of MCs (10, 14).

In this study, we evaluated MC burden by measuring bST and percentages of MC in the BM aspirate of distinct patients’ groups and control individuals. In line with other investigations (26–29), we observed a higher MC burden in CMCD as compared to patients without a CMCD. However, there was no difference in MC burden and KIT D816V mutational burden between CMCD patients with or without wasp venom anaphylaxis. This reinforces earlier observations that the MC burden only partially explains the difference in anaphylactic risk between CMCD patients and non-CMCD patients, but not the occurrence of anaphylaxis within the CMCD population (9). It suggests that other factors like the intrinsic properties of MCs likely contribute to the risk for anaphylaxis in CMCD (30–32).

It is known that MCs from patients with a non-advanced CMCD exhibit an aberrant active phenotype with lower surface expression of CD117 and overexpression of the activation markers CD203c and CD63 (33, 34). Our findings go beyond a confirmation of this aberrant active MC phenotype in patients with a CMCD. We show that MCs in CMCD also overexpress the inhibitory receptor CD300a as compared to patients without a CMCD. The exact reason(s) for this overexpression remain(s) uncertain. It has been demonstrated that CD300a can be upregulated in response to both IgE- dependent and IgE-independent activation and participates in a self-limiting process through cis-interaction with phosphatidylserine externalized on the cell (35, 36). Most importantly, it also has been shown that CD300a inhibits IgE-dependent cell activation in MC and basophils (37–39). It is tempting to speculate that the overexpression of CD300a is an attempt of the MC to dampen its aberrant activated status. Together with the increased MC burden, the overexpression of CD203c, CD63 and CD300a likely reflects an aberrant spontaneous activated status and an enhancement of effector functions of MCs in patients with a CMCD (33, 40). The spontaneous activated status of MCs in patients with a CMCD could result from the higher density of surface FcεRI on the aberrant MCs. A higher density of FcεRI could facilitate intracellular signaling mediated by spontaneous aggregation of the FcεRI that are occupied with IgE (41–43).

In our study, the main differentiating factor between CMCD patients with wasp venom anaphylaxis and CMCD patients without anaphylaxis is the increased surface expression of FcεRI by MCs. *In vitro* studies have shown that MCs with upregulated FcεRI expression are significantly more sensitive to activation and that this enhanced the degranulation and production of cytokines and lipid mediators (44, 45). When FcεRIs are in closer proximity to each other, cross-linking of the sIgE/FcεRIs complexes by an appropriate allergen leads to clustering of higher number of sIgE/FcεRI complexes on the MCs. This enhanced clustering propagates a stronger signal for degranulation of MCs (41, 42). These *in vitro* data are reinforced by a clinical study of patients with allergic rhinitis treated with omalizumab. In this study, treatment with the monoclonal anti-IgE antibody omalizumab decreased MC FcεRI expression and mitigated allergen-induced skin test responsiveness (46). FcεRI expression is highly dependent on the occupancy of IgE that stabilizes the receptor and prevents degradation and internalization (47). The higher level of tIgE in CMCD patients with wasp venom anaphylaxis compared to CMCD patients without anaphylaxis can explain the higher expression of FcεRI in the wasp venom anaphylaxis group. Important to mention, the number of atopic patients is higher in the group of CMCD patients with anaphylaxis compared to CMCD patients without anaphylaxis which can also influence the difference in tIgE. However, the level of tIgE cannot explain the differences found between patients with and without CMCD. In our study, the expression of FcεRI was primarily determined by the clonality of the MCs rather than the levels of tIgE. This is reflected by the fact that MCs from patients with wasp venom anaphylaxis, who had similar levels of tIgE as patients with wasp venom anaphylaxis and CMCD, showed a lower surface expression of FcεRI. The data from patients with both an aberrant and non-aberrant MC subpopulation confirm this finding. Here the aberrant MCs had a higher expression of FcεRI than their non-aberrant counterparts while being exposed to the same amounts of tIgE. Besides, it is known that the level of FcεRI expression in CMCD depends on the level of mast cell maturity. Of note the expression of FcεRI in patients with CMCD is higher than in patients with WVA without CMCD that display non aberrant mature mast cells. In this study, CMCD patients with anaphylaxis without IgE sensitization are not included. Consequently, we cannot exclude that sensitization to wasp in combination with CMCD may have a synergistic effect on the expression of FcεRI. However, we would like to highlight that the number of these patients is low as most of the CMCD patients with anaphylaxis experienced an IgE-mediated reaction (48).

Previous studies have shown that MCs from patients with CMCD are more frequently localized perivascularly as compared to control population (49). Although not investigated in this study, we can hypothesize that perivascular MCs capture IgE through active probing of the blood vessel (50). Consequently because of their localization they might have a greater influence on the vascular permeability when releasing their vasoactive mediators in wasp venom anaphylaxis. The active probing of blood vessels combined with the perivascular mediator release by these MCs might explain the typical pattern of wasp venom anaphylaxis that is dominated by hypotension when exposed to a sensitized antigen (51).

It has been hypothesized that in patients with CMCD, IgE/FcεRI-independent signaling might contribute to MC activation (13). Moreover, it has been proposed that the MRGPRX2 activation by mastoparan might also contribute to wasp venom anaphylaxis in SM (11, 13, 52–60). We did not find significant differences in MRGPRX2 expression between the different study populations. These findings seem to indicate that MRGPRX2 on bone marrow MCs is unlikely to play a major role in WVA, nor does its expression advance prediction of the occurrence of wasp venom anaphylaxis within the CMCD population. Admittedly, the functionality of MRGPRX2 remains to be elaborated, especially as polymorphisms of mutations might influence the ability of interaction with ligands as mastoparan (61). Moreover, one can speculate that there are different expression patterns depending on the localization of the MC. In fact, we cannot exclude that upregulation of MRGPRX2 on skin MCs, as recently investigated in lesions of MIS (62), might play a role in wasp venom anaphylaxis.

Furthermore, in this study we demonstrate that the MC phenotype of patient with an elevated baseline serum tryptase mostly segregates with the phenotype of patients with a non-clonal disease and that there is no common marker expression pattern in this population that would predict increased susceptibility to wasp venom anaphylaxis.

Basophils are nonredundant effector cells in wasp venom anaphylaxis (14, 63). Therefore, the phenotype and functionality of basophils can influence the clinical phenotype. Consequently, we also aimed at studying the intrinsic properties of basophils in our different study populations. However, there is no difference in phenotype and functionality, this strongly suggests that MCs, rather than basophils, account for the differences in occurrence/risk of anaphylaxis between the different study groups.

In conclusion, the expression of FcεRI on MCs, but not the quantitative expression of MRGPRX2 or the phenotype and function of basophil, plays a crucial role in determining the risk of wasp venom anaphylaxis in patients with non-advanced CMCD. This high expression is not strictly related to the total IgE but is an intrinsic characteristic of the activated status of aberrant MCs. Given the overexpression of FcεRI on MCs in CMCD patients with anaphylaxis, this might be a potential therapeutic target.

## Data Availability Statement

The raw data supporting the conclusions of this article will be made available by the authors, without undue reservation.

## Ethics Statement

The studies involving human participants were reviewed and approved by the Ethics committee of the Antwerp University Hospital (B300201836890). The patients/participants provided their written informend consent to participate in this study.

## Author Contributions

LP and JE designed the study, performed all experiments and analysis, and wrote the first draft of the paper. The experiments were performed under supervision of CB, CM, and MH who also contributed to the experimental design. DE and VS coordinated and supervised the project and contribute to writing the paper. All authors contributed to the article and approved the submitted version.

## Funding

The Research Foundation Flanders/Fonds Wetenschappelijk Onderzoek (FWO: 1804518N).

## Conflict of Interest

The authors declare that the research was conducted in the absence of any commercial or financial relationships that could be construed as a potential conflict of interest.

The reviewer RMC declared a past co-authorship with the authors DE, MF, AG, MMH, CB, ID, and VS to the handling Editor.

## Publisher’s Note

All claims expressed in this article are solely those of the authors and do not necessarily represent those of their affiliated organizations, or those of the publisher, the editors and the reviewers. Any product that may be evaluated in this article, or claim that may be made by its manufacturer, is not guaranteed or endorsed by the publisher.

## Supplementary Material

Supplementary Figure 1 Gating strategy of bone marrow mast cells and peripheral blood basophils. Single cells were selected based on FSC-A and FSC-H. The first step in the mast cell gating is selecting the leukocytes based on SSC and CD45. Only the leukocytes expressing CD117 were selected. Mast cells were defined as CD45^+^CD117^++^CD203c^+^. Basophils were selected as low side scatter and IgE^+^ and CD203c^+^. Fluorescence minus one (FMO) samples were used to set a marker between positive and negative cells according to the 99^th^ percentile.

Supplementary Figure 2 Representative plots of immunophenotyping of bone marrow mast cells with corresponding fluorescence minus one samples. Staining of membrane markers on bone marrow mast cells from two different patients. Fluorescence minus one (FMO) samples were used to set a marker between positive and negative cells according to the 99th percentile. The markers are set interindividual.

Supplementary Figure 3 Titer of specific IgE to wasp venom and ratio of specific to total IgE ratio. In the patients with wasp venom anaphylaxis (with and without a CMCD), there was no significant difference between sIgE and the sIgE-to-tIgE ratios. CMCD/WVA^+^ = Patients with a clonal mast cell disorder and wasp venom anaphylaxis. WVA^+^ = Patients with wasp venom anaphylaxis and without clonal mast cell disorder.

Supplementary Figure 4 Activation of peripheral blood basophils with anti-IgE or fMLP and coincubations with various concentrations of SCF. There is no difference in the upregulation of CD203c and CD63 between the different study population after stimulation with anti-IgE and fMLP. Coincubations with various concentrations of SCF did not alter responsiveness of the cells neither to anti-IgE, nor to fMLP. CMCD/WVA^+^ = Patients with a clonal mast cell disorder and wasp venom anaphylaxis. CMCD/ANA^-^= Patients with a clonal mast cell disorder without anaphylaxis. WVA^+^ = Patients with wasp venom anaphylaxis and without clonal mast cell disorder. EBST = patients with an elevated baseline serum tryptase without anaphylaxis. HC = Healthy controls. fMLP = f-Met-Leu-Phe. SCF = Stem cell factor.

## Abbreviations

BM, Bone marrow; BSA, Bovine Serum Albumin; bST, Baseline serum tryptase; CMCD(s), Clonal mast cell disorder(s); EDTA, Ethylenediaminetetraacetic acid; FEIA, Fluorescence Enzyme Immunoassay; fMLP, f-Met-Leu-Phe; HC(s), Healthy control(s); EBST, Elevated baseline serum tryptase; ISM, Indolent systemic mastocytosis; MC(s), Mast cell(s); MESF, Molecules of Equivalent Soluble Fluorochrome; MIS, Mastocytosis in the skin; MMAS, Monoclonal mast cell activation syndrome; MRGPRX2, Mas-related G protein coupled receptor; NMHP, Non-mast cell related haematological pathology; NS, Non-significant; PB, Peripheral blood; PBS, Phosphate-buffered saline; rVes v, Recombinant Vespula vulgaris; SCF, Stem cell factor; sIgE, Specific IgE; SM, Systemic mastocytosis; tIgE, Total IgE; v-sIgE, Venom-specific IgE antibodies; WVA, Wasp venom anaphylaxis.
