# Supplementary figures and images for: Overexpression of FcεRI on Bone Marrow Mast Cells, but Not MRGPRX2, in Clonal Mast Cell Disorders With Wasp Venom Anaphylaxis
Source: Front Immunol. 2022 Feb 25;13:835618. doi: 10.3389/fimmu.2022.835618 (PMC8914951; doi:10.3389/fimmu.2022.835618)

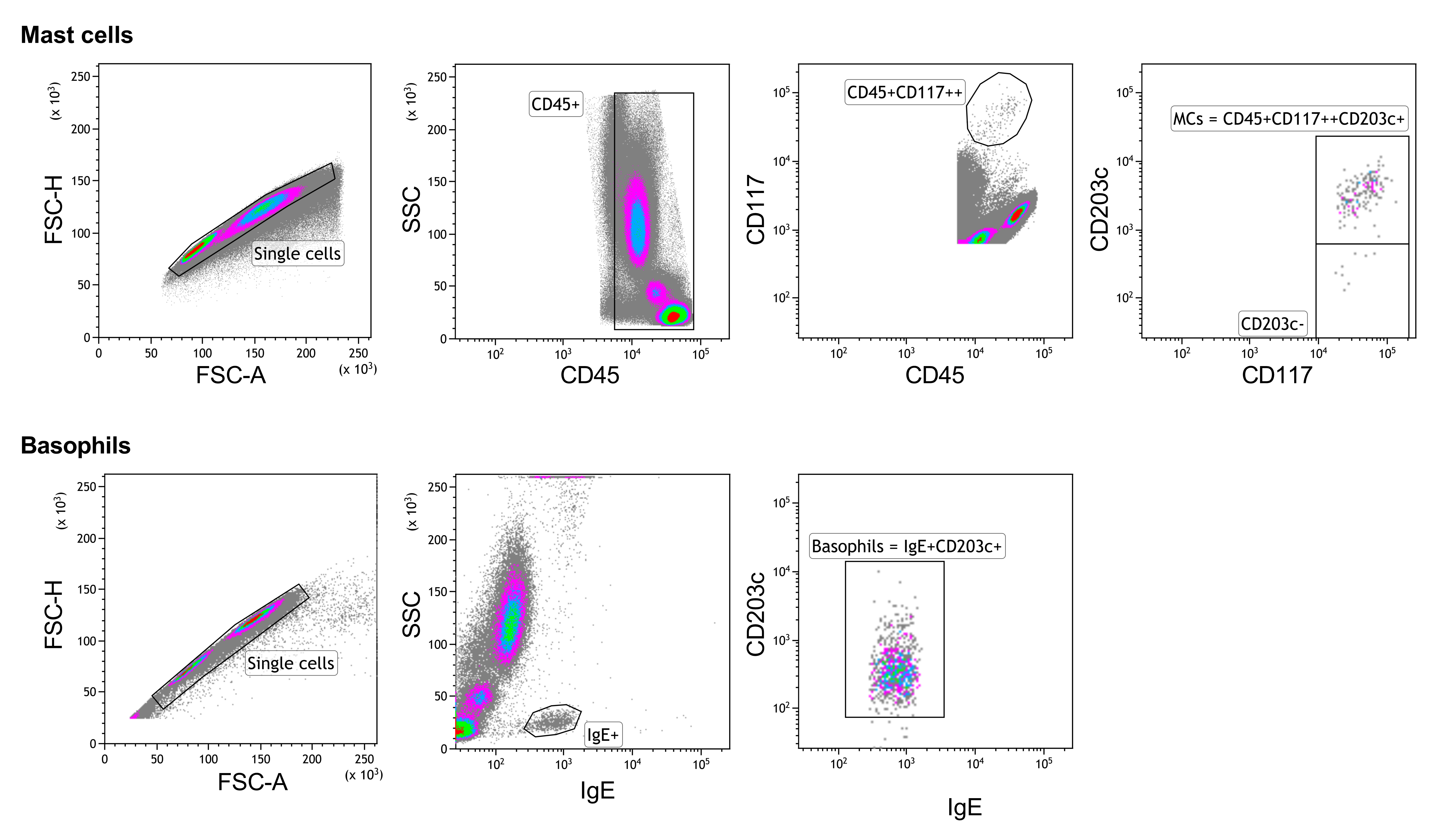

Supplement: Supplementary Figure 1 — Gating strategy of bone marrow mast cells and peripheral blood basophils. Single cells were selected based on FSC-A and FSC-H. The first step in the mast cell gating is selecting the leukocytes based on SSC and CD45. Only the leukocytes expressing CD117 were selected. Mast cells were defined as CD45+CD117++CD203c+. Basophils were selected as low side scatter and IgE+ and CD203c+. Fluorescence minus one (FMO) samples were used to set a marker between positive and negative cells according to the 99th percentile. [file Image_1.tif]

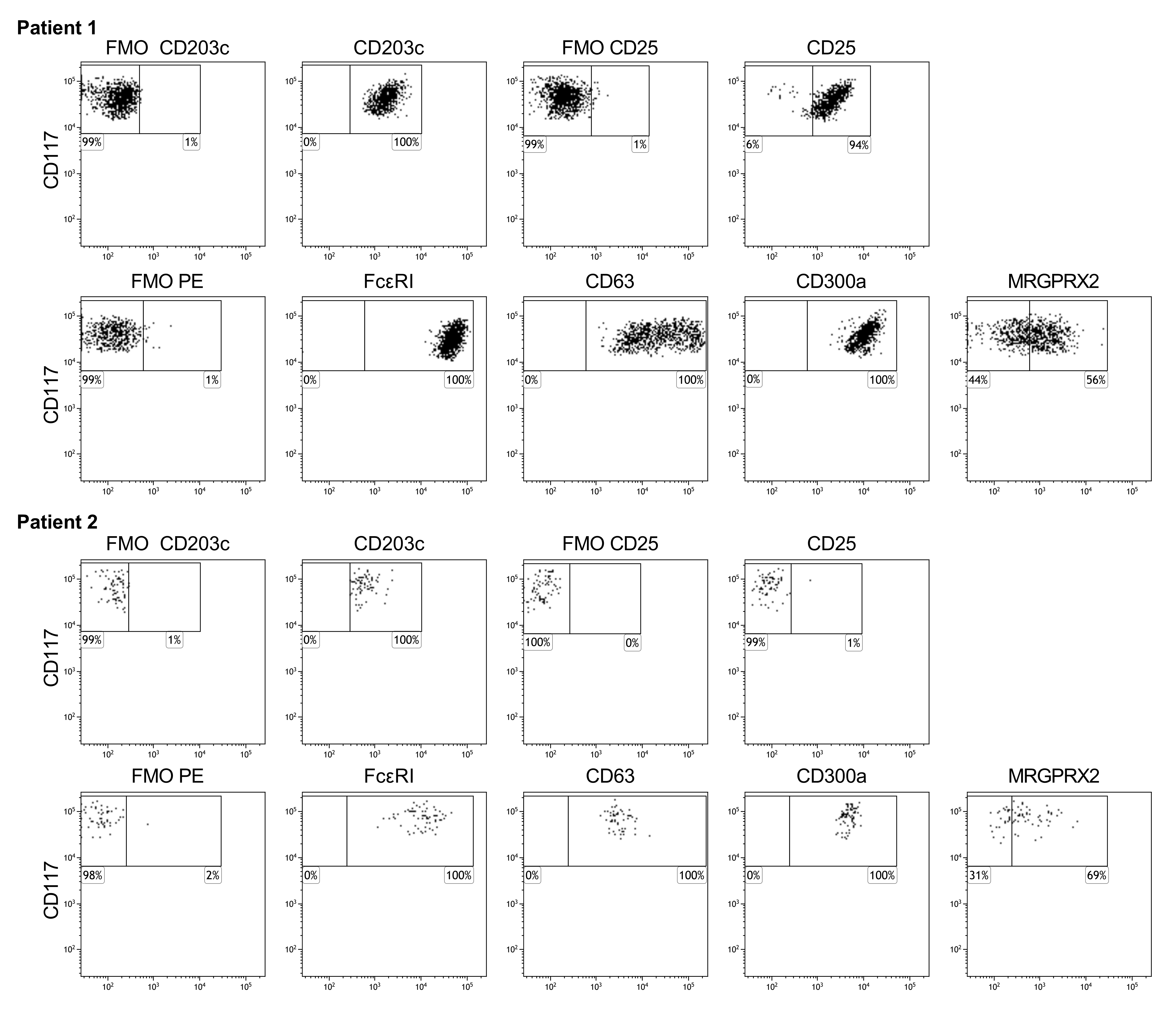

Supplement: Supplementary Figure 2 — Representative plots of immunophenotyping of bone marrow mast cells with corresponding fluorescence minus one samples. Staining of membrane markers on bone marrow mast cells from two different patients. Fluorescence minus one (FMO) samples were used to set a marker between positive and negative cells according to the 99th percentile. The markers are set interindividual. [file Image_2.tif]

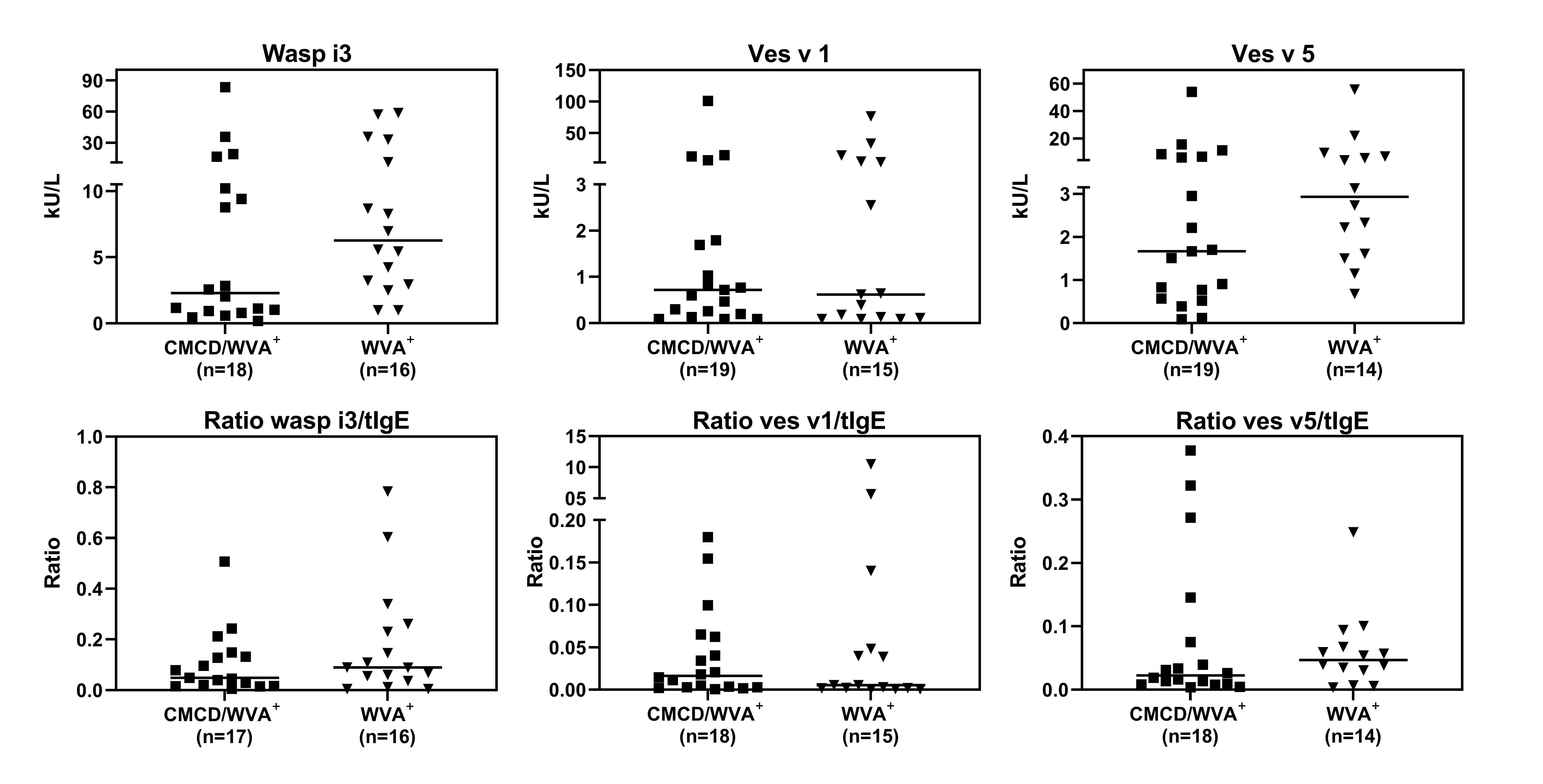

Supplement: Supplementary Figure 3 — Titer of specific IgE to wasp venom and ratio of specific to total IgE ratio. In the patients with wasp venom anaphylaxis (with and without a CMCD), there was no significant difference between sIgE and the sIgE-to-tIgE ratios. CMCD/WVA+ = Patients with a clonal mast cell disorder and wasp venom anaphylaxis. WVA+ = Patients with wasp venom anaphylaxis and without clonal mast cell disorder. [file Image_3.tif]

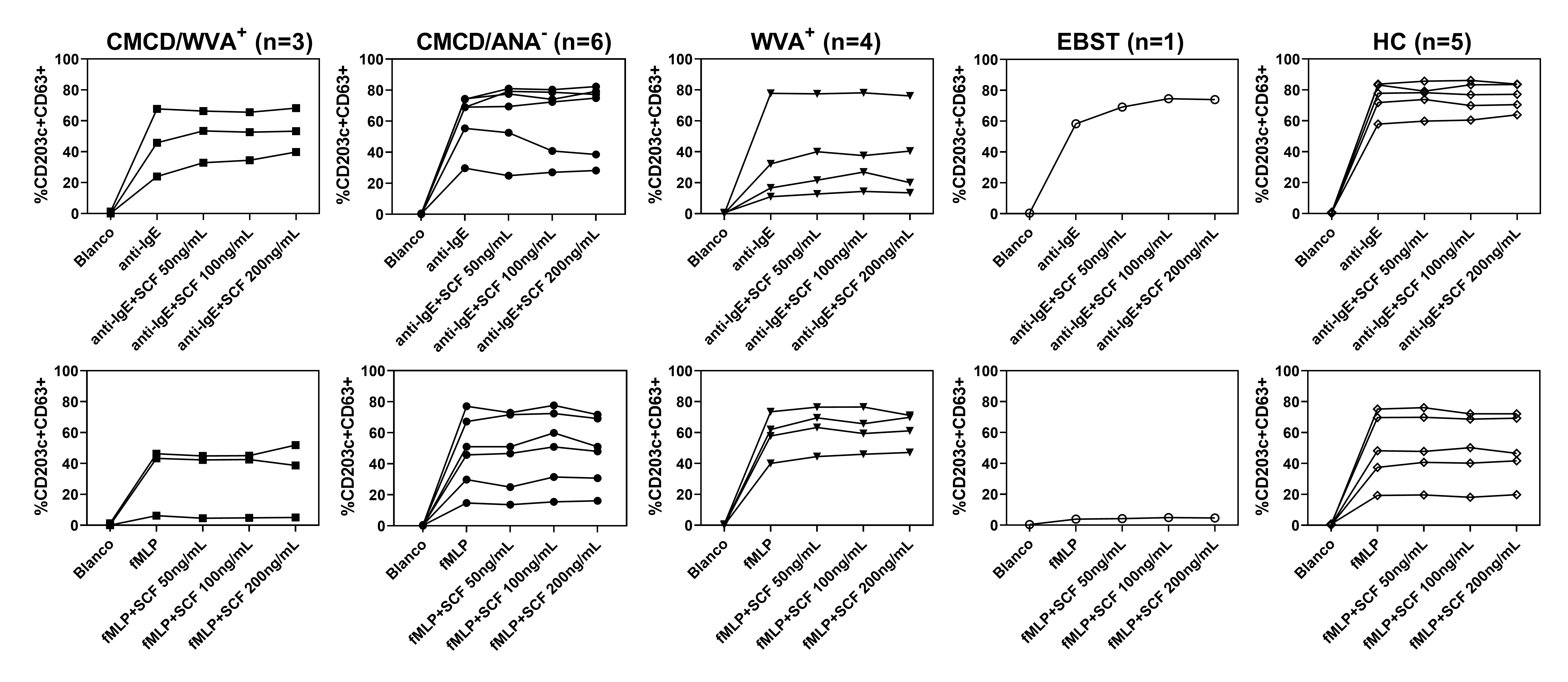

Supplement: Supplementary Figure 4 — Activation of peripheral blood basophils with anti-IgE or fMLP and coincubations with various concentrations of SCF. There is no difference in the upregulation of CD203c and CD63 between the different study population after stimulation with anti-IgE and fMLP. Coincubations with various concentrations of SCF did not alter responsiveness of the cells neither to anti-IgE, nor to fMLP. CMCD/WVA+ = Patients with a clonal mast cell disorder and wasp venom anaphylaxis. CMCD/ANA-= Patients with a clonal mast cell disorder without anaphylaxis. WVA+ = Patients with wasp venom anaphylaxis and without clonal mast cell disorder. EBST = patients with an elevated baseline serum tryptase without anaphylaxis. HC = Healthy controls. fMLP = f-Met-Leu-Phe. SCF = Stem cell factor. [file Image_4.tif]
